# Aplidin (plitidepsin) is a novel anti-myeloma agent with potent anti-resorptive activity mediated by direct effects on osteoclasts

**DOI:** 10.18632/oncotarget.26831

**Published:** 2019-04-12

**Authors:** Jesus Delgado-Calle, Noriyoshi Kurihara, Emily G. Atkinson, Jessica Nelson, Kazuaki Miyagawa, Carlos Maria Galmarini, G. David Roodman, Teresita Bellido

**Affiliations:** ^1^ Department of Medicine, Division of Hematology/Oncology, Indiana University School of Medicine, Indianapolis, IN, USA; ^2^ Department of Anatomy and Cell Biology, Indiana University Sc hool of Medicine, Indianapolis, IN, USA; ^3^ Department of Medicine, Division of Endocrinology, Indiana University School of Medicine, Indianapolis, IN, USA; ^4^ Indiana Center for Musculoskeletal Health, Indiana University School of Medicine, Indianapolis, IN, USA; ^5^ Research Department, PharmaMar S.A., Madrid, Spain; ^6^ Roudebush VA Medical Center, Indianapolis, IN, USA

**Keywords:** myeloma, osteoclasts, osteocytes, osteoblasts, tumor

## Abstract

Despite recent progress in its treatment, Multiple Myeloma (MM) remains incurable and its associated bone disease persists even after complete remission. Thus, identification of new therapeutic agents that simultaneously suppress MM growth and protect bone is an unmet need. Herein, we examined the effects of Aplidin, a novel anti-cancer marine-derived compound, on MM and bone cells. In vitro, Aplidin potently inhibited MM cell growth and induced apoptosis, effects that were enhanced by dexamethasone (Dex) and bortezomib (Btz). Aplidin modestly reduced osteocyte/osteoblast viability and decreased osteoblast mineralization, effects that were enhanced by Dex and partially prevented by Btz. Further, Aplidin markedly decreased osteoclast precursor numbers and differentiation, and reduced mature osteoclast number and resorption activity. Moreover, Aplidin reduced Dex-induced osteoclast differentiation and further decreased osteoclast number when combined with Btz. Lastly, Aplidin alone, or suboptimal doses of Aplidin combined with Dex or Btz, decreased tumor growth and bone resorption in *ex vivo* bone organ cultures that reproduce the 3D-organization and the cellular diversity of the MM/bone marrow niche. These results demonstrate that Aplidin has potent anti-myeloma and anti-resorptive properties, and enhances proteasome inhibitors blockade of MM growth and bone destruction.

## INTRODUCTION

Multiple myeloma (MM) is the second most common hematologic malignancy and the most frequent cancer that involves bone [1, 2]. The bone/bone marrow (BM) niche plays a critical role in MM onset and progression. MM cells locate in specialized niches in the BM where they interact with stromal cells, endothelial cells, immune cells, osteoblasts, osteoclasts, adipocytes, and osteocytes [2, 3]. These interactions transform the bone/BM niche into an ideal environment for the proliferation and survival of MM cells. Further, actively expanding MM cells increase osteoclast numbers and activity, leading to development of lytic bone lesions that rarely heal due to a concomitant suppression of osteoblast bone forming activity [1, 4]. MM can cause pathologic fractures and excruciating bone pain, which increase morbidity and mortality and diminish the quality of life of patients. Remarkable progress has been made in the treatment of MM in the past 10 years, however MM remains incurable in the majority of patients. In addition, osteolytic lesions persist in patients in remission with no evidence of marrow infiltration by MM cells [4, 5], thus prompting a continued search for additional therapeutic options that inhibit MM growth and protect bone health.

Aplidin is a novel anti-cancer compound isolated from the marine tunicate *Aplidium albicans* [6–8]. *In vitro* studies showed that Aplidin has anti-MM activity against 19 MM cell lines including cells resistant to anti-MM agents frequently used in the clinic (i.e. melphalan, doxorubicin, thalidomide derivatives, and dexamethasone) and primary MM cells isolated from patients (13 out 16 showed response to Aplidin) [9]. Recently, Losada et al demonstrated that Aplidin targets the eukaryotic elongation factor 1A2 (EF1A2), which is overexpressed in MM cells [7]. Mechanistically, several pathways have been identified to mediate the effects of Aplidin on the viability of MM cells. Aplidin induces apoptosis in MM cells, which involves activation of p38 and c-jun NH(2)-terminal kinase signaling, Fas/CD95 translocation to lipid rafts, and ultimately caspase activation. In addition, Aplidin decreases the proliferation of MM cells, an effect mediated by the suppression of several proliferative genes. [9, 10]. *In vivo*, it has been reported that Aplidin has anti-MM activity in preclinical animal models [9, 10]. Further, in a pivotal phase III trial of patients with relapsed or refractory MM, Aplidin in combination with dexamethasone (Dex) significantly reduced the risk of progression or death compared to Dex alone [11]. However, the effects of Aplidin, alone or in combination with anti-MM therapy, on bone cells and MM-induced bone disease are unknown.

In this study, we examined the effects of Aplidin, alone or in combination with other anti-MM drugs, on MM cells and bone cells (osteocytes, osteoblasts, and osteoclasts). Using *in vitro* approaches and an *ex vivo* 3D model of MM bone disease, we found that Aplidin decreased MM cell viability, and that this action was enhanced by the anti-MM drugs Dex and Bortezomib (Btz). In addition, Aplidin modestly decreased osteocyte and osteoblast viability, and this effect was exacerbated by Dex, but partially prevented by Btz. Importantly, Aplidin potently inhibited osteoclast precursor commitment and differentiation, inhibited mature osteoclast bone resorption, and reduced Dex-induced increases in osteoclast differentiation. These findings demonstrate that Aplidin inhibits both tumor growth and bone resorption, and suggest that Aplidin can enhance the clinical efficacy of proteasome inhibitors by potentiating their anti-tumor properties and reducing the risk of skeletal-related events by inhibiting resorption through acting on osteoclasts.

## RESULTS

### The anti-myeloma effects of Aplidin are enhanced by dexamethasone and bortezomib

**Figure 1:**
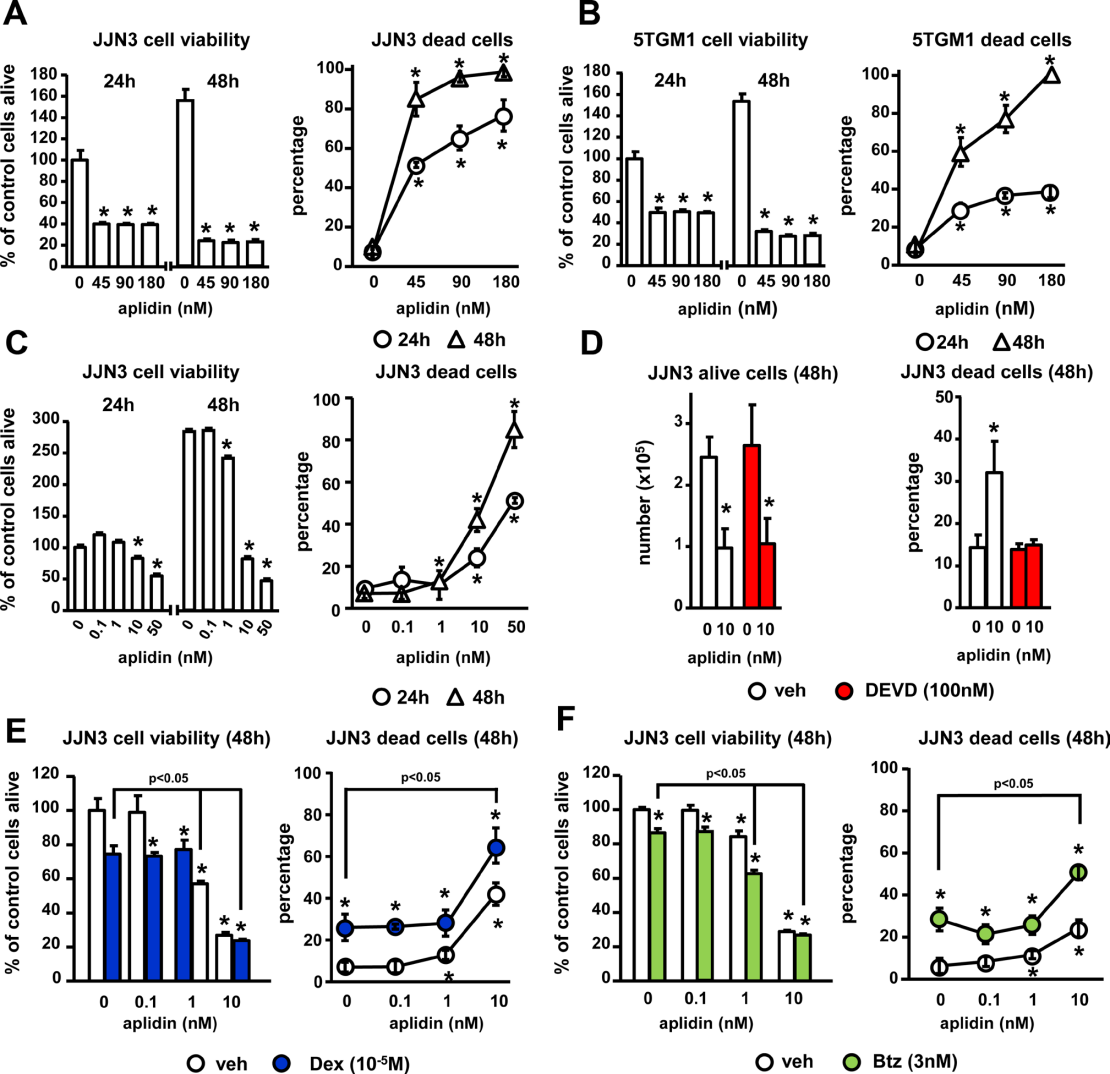
The inhibition of MM cell viability by Aplidin is enhanced by Dex and Btz (**A**–**C**) Human JJN3 and murine 5TGM1 MM cells were treated with increasing concentrations of Aplidin and MM cell viability/death was evaluated after 24 h and 48 h using MTT and Trypan blue uptake assays. JJN3 MM cells were treated with Aplidin 10 nM with/without DEVD (**D**), and increasing concentrations of Aplidin in the presence/absence of a fixed dose of Dex (**E**) or Btz (**F**) and cell viability/death was evaluated after 48 h. Representative experiments out of two are shown (*n* = 4–6 per condition). Bars represent means ± SD. ^*^p < 0.05 vs vehicle; lines indicate *p* < 0.05 for Dex/Btz alone vs Dex/Btz + Aplidin.

We first determined the dose- and time-dependent effects of Aplidin on the viability of murine and human MM cell lines. Concentrations higher than 1 nM of Aplidin decreased the viability of human JJN3 MM cells in a dose-dependent manner (EC50~10 nM) and progressively reduced MM cell viability from 24 h to 48 h (Figure 1A and 1C). Aplidin also decreased the viability of murine 5TGM1 MM cells (Figure 1B). Aplidin induced MM cell death in a dose and time dependent manner in both JJN3 and 5TGM1 MM cells (Figure 1A and 1B), with an EC_50_ of ~10nM Aplidin for JJN3 MM cells and ~20 nM for 5TGM1 cells after 48 h of treatment (Figure 1C), and decreased the proliferation of JJN3 MM cells (Figure 1D). The elevated MM cell death induced by Aplidin was due to apoptosis, as treatment with the caspase 3 inhibitor DEVD fully prevented Aplidin-induced increases in MM cell death (Figure 1D). In contrast, DEVD did not affect the number of alive MM cells, which remained decreased by Aplidin (Figure 1D).

We next evaluated the effects of combinations of Aplidin with other anti-MM drugs on MM cell viability/cell death. Treatment with Dex alone decreased the viability of JJN3 cells, increased MM cell death up to 23%, and enhanced the effect of 10 nM Aplidin on MM cell death by 1.6-fold (39% vs 63% cell death, Aplidin vs Aplidin+Dex, respectively; Figure 1E). Btz also decreased JJN3 viability by 50%, augmented JJN3 cell death up to 35%, and increased the number of JJN3 dead cells in combination with 10 nM Aplidin by 2.5-fold (20% vs 50% cell death, Aplidin vs Aplidin+Btz, respectively; Figure 1F). These results demonstrate that Aplidin induces MM cell death and its anti-MM activity is further enhanced when combined with Dex and Btz.

### Aplidin-mediated decrease in osteocyte viability is enhanced by dexamethasone and partially prevented by bortezomib

**Figure 2:**
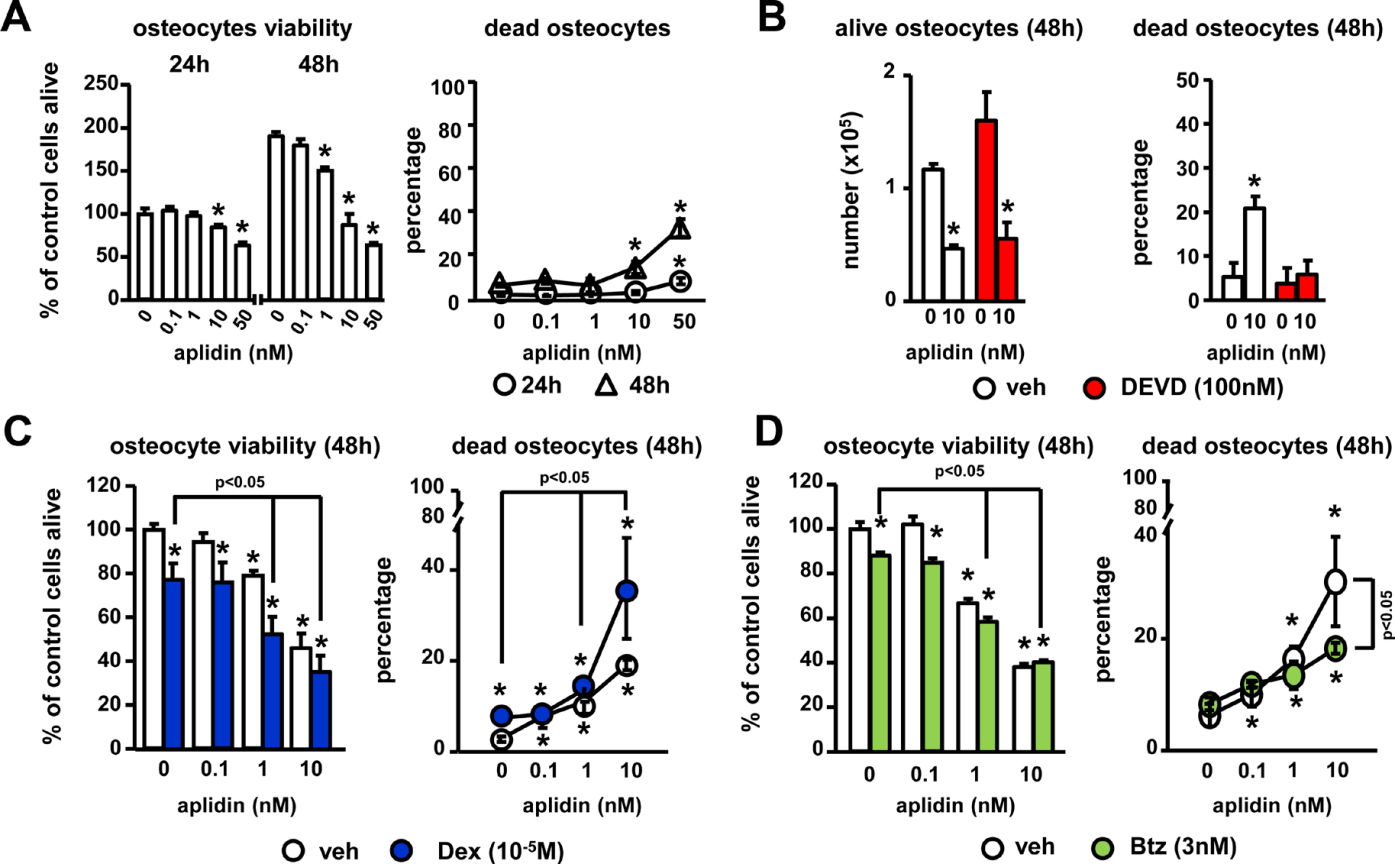
Aplidin decreases osteocyte viability (**A**) Time (24–48 h) and dose-response (0–10 nM) to Aplidin in murine MLO-A5 osteocyte-like cells. Evaluation of the effects of combinations of Aplidin with the caspase 3 inhibitor DEVD (**B**), Dex (**C**) and Btz (**D**) after 48 h treatment. Representative experiments out of two are shown (*n* = 4–6 per condition). Bars represent means ± SD. ^*^p < 0.05 vs vehicle; lines indicate *p* < 0.05 for Dex/Btz alone vs Dex/Btz + Aplidin, and Aplidin vs Aplidin + Btz.

To gain insight into the effects of Aplidin in the skeleton, we first investigated its effects on osteocytes, the most abundant cells in bone. Aplidin 10nM increased the percentage of dead MLO-A5 osteocyte-like cells up to 10–15% after 48 h (Figure 2A), and inhibit MLO-A5 osteocyte-like proliferation (Figure 2A, 2B). Mechanistic studies showed that Aplidin increases osteocyte cell death by inducing apoptosis. As shown in Figure 2B, treatment with the caspase 3 inhibitor DEVD fully blocked Aplidin-induced increases in osteocyte cell death; however, treatment with DEVD did not prevent the decreased in the number of alive osteocytes induced by Aplidin. Further, as shown previously [12], treatment with Dex increased osteocyte cell death to ~8%, and in combination with Aplidin 10 nM, Dex further increased Aplidin-induced osteocyte cell death by 1.8-fold (19% vs 37% cell death, Aplidin vs Aplidin+Dex, respectively; Figure 2C). In contrast, Btz did not alter osteocyte cell death and partially blunted the increase in dead osteocytes induced by Aplidin 10 nM (31% vs 19%, Aplidin vs Aplidin+Btz, respectively) (Figure 2D).

### Co-administration of bortezomib partially prevents the decreases in osteoblast viability and function induced by Aplidin

**Figure 3:**
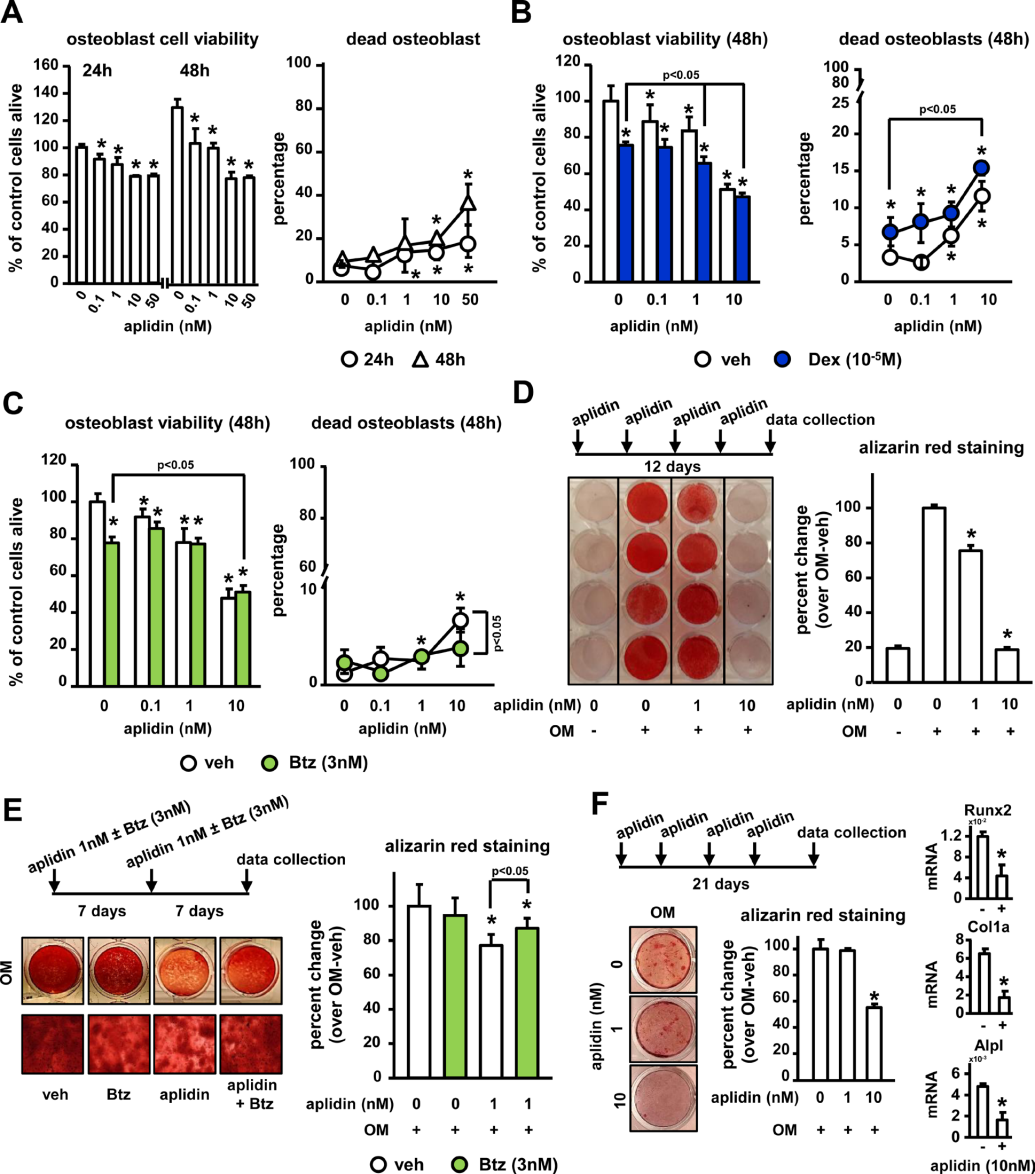
Aplidin decreases osteoblast viability and function and co-administration of Btz partially prevents these effects (**A**–**C**) Cell viability and cell death was evaluated in murine OB-6 osteoblastic cells treated with Aplidin (0–10 nM) alone or in combination with Dex or Btz. Representative experiments out of two are shown (*n* = 4–6 per condition). Mineralization of OB-6 (**D**–**E**) and MC-4 (**F**) cells treated with/without Aplidin in the presence/absence of Btz for 14-21 days evaluated by Alizarin Red staining. Percent change was calculated over cells cultured with osteogenic media (OM) in the absence of Aplidin. Representative experiments out of two are shown (*n* = 3–4 per condition). Bars represent means ± SD. ^*^p < 0.05 vehicle; lines indicate *p* values < 0.05 comparing Dex/Btz alone vs Dex/Btz + Aplidin, and Aplidin vs Aplidin + Btz.

We next examined the actions of Aplidin on osteoblasts. Concentrations of 1 nM Aplidin and higher increased cell death up to 10–20% after 48 h in OB-6 osteoblastic cells (Figure 3A). As previously demonstrated, Dex increased osteoblast cell death, and when combined with Aplidin 1 and 10 nM, it further enhanced this effect (10% vs 15%, Aplidin 10 nM vs Aplidin 10 nM+Dex, respectively) (Figure 3B). As observed in osteocytes cultures, Btz did not alter the viability of osteoblasts; however, it partially reduced the increases in the number of dead osteoblasts induced by 10 nM Aplidin (8% vs 3%, Aplidin 10 nM vs Aplidin 10 nM+Dex, respectively) (Figure 3C).

Continuous (5 days a week) and pulse (once a week) administration of 1–10 nM Aplidin decreased the matrix production and mineralization of OB-6 cells cultured under osteogenic conditions by 20% (Figure 3D and 3E). Further, consistent with the protective effects of Btz against Aplidin-induced osteoblast cell death, co-administration of Btz partially prevented (~50%) the reduction in mineralization induced by Aplidin 1 nM (Figure 3E). Similar to the findings in OB-6 cells, continuous treatment with Aplidin 10 nM decreased matrix mineralization in pre-osteoblastic MC4 cells (Figure 3F). This effect was associated with decreases in the mRNA expression of Runx2, Collagen 1a, and alkaline phosphatase (Figure 3F).

### Aplidin inhibits osteoclast precursor differentiation and the bone resorbing activity of mature osteoclasts

**Figure 4:**
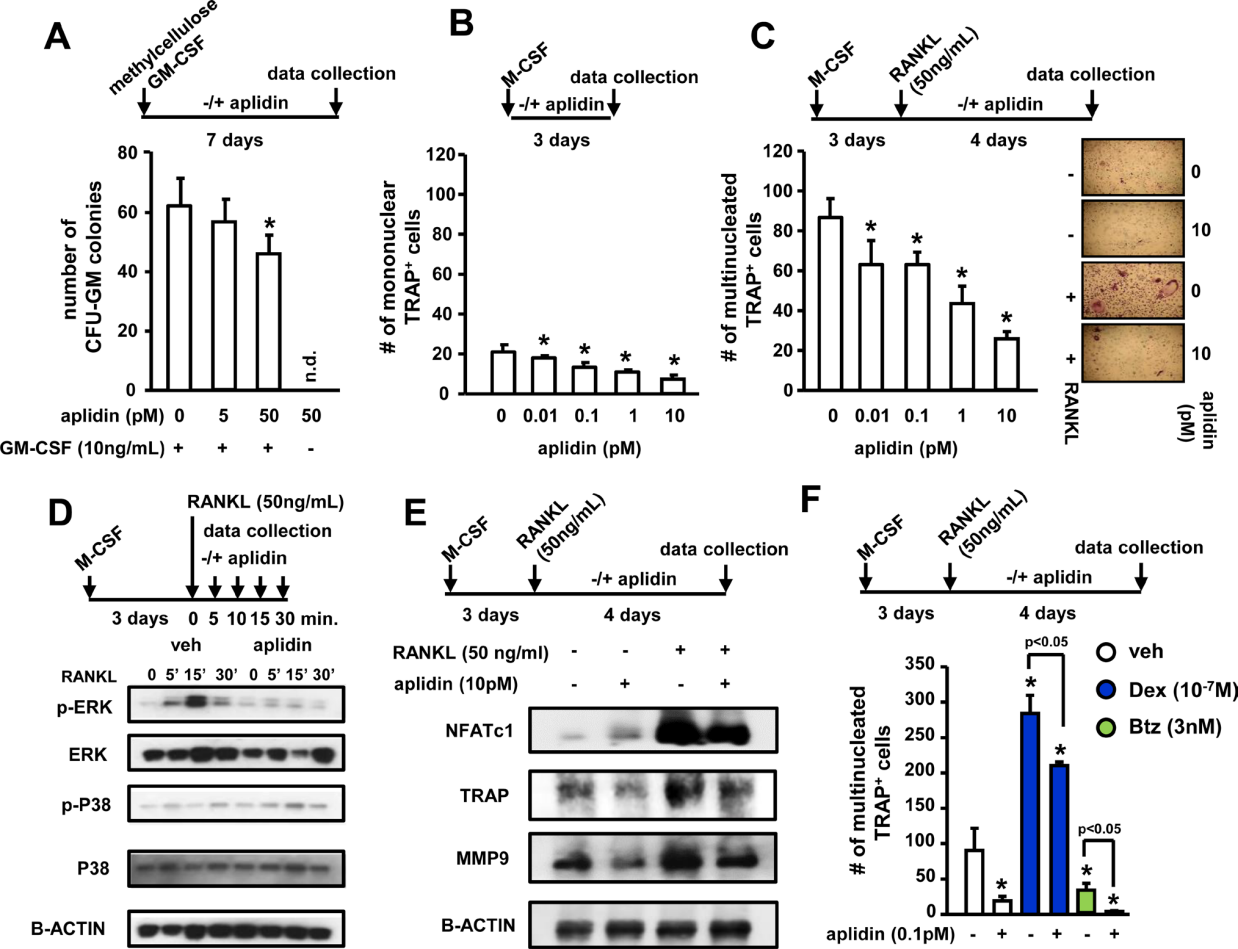
Aplidin inhibits osteoclast precursor expansion and differentiation (**A**) Evaluation of the effects of Aplidin (0–50 pM) on proliferation and differentiation of hematopoietic progenitors (CFU-GM), (**B**) expansion of the osteoclast precursor population induced by M-CSF, and (**C**) RANKL-induced differentiation of osteoclast precursors. Representative images of osteoclast cultures are shown in panel C. (**D**) Analysis of protein levels of p-ERK, ERK, p-P38, and P38 in cell lysates collected from osteoclast precursors stimulated with RANKL (0–30 minutes). (**E**) Analysis of the protein levels of NFATc1, TRAP, and MMP9. (**F**) Number of TRAP+ osteoclasts after treatment with Aplidin in the presence/absence of Dex and Btz. Representative experiments out of two are shown (*n* = 4 per condition). Bars represent means ± SD. ^*^p < 0.05 vs vehicle; lines indicate *p* values < 0.05 comparing Dex/Btz alone vs Dex/Btz + Aplidin.

We also investigated the effects of Aplidin on osteoclast formation and mature osteoclasts. *In vitro*, osteoclast lineage cells exhibited higher sensitivity to Aplidin as compared to MM cells or cells of the osteoblastic lineage, as nM concentrations of Aplidin fully blocked the formation of CFU-GM colonies and osteoclast formation. Thus, the drug was tested at lower concentrations. 50 pM Aplidin decreased by 15% the number of CFU-GM colonies in cultures of murine bone marrow cells (Figure 4A). Moreover, concentrations as low as 0.01 pM of Aplidin reduced TRAP positive mononuclear osteoclast precursors by 10% (Figure 4B). In addition, Aplidin decreased the number of multinucleated osteoclasts by 40% (Figure 4C). Aplidin blunted the rapid (5–15 min) increase in ERK phosphorylation induced by RANKL, but did not affect RANKL-induced p38 phosphorylation (Figure 4D), suggesting that Aplidin inhibited RANKL-induced differentiation of osteoclast precursors by preventing ERK activation. Treatment with Aplidin also decreased the RANKL-mediated induction of markers of osteoclast differentiation, including NFATc1, TRAP, and MMP-9 (Figure 4E). Further, treatment with Dex induced a 3.3-fold increase in the number of osteoclasts induced by RANKL, whereas Btz decreased the differentiation of osteoclast precursors by ~50% compared to vehicle-treated cells (Figure 4F). Addition of Aplidin to Dex treated marrow cultures resulted in a 25% reduction in the increase of TRAP+ multinucleated cells induced by Dex, and further decreased the number of osteoclasts in pre-osteoclast cultures treated with Btz (Figure 4F).

**Figure 5:**
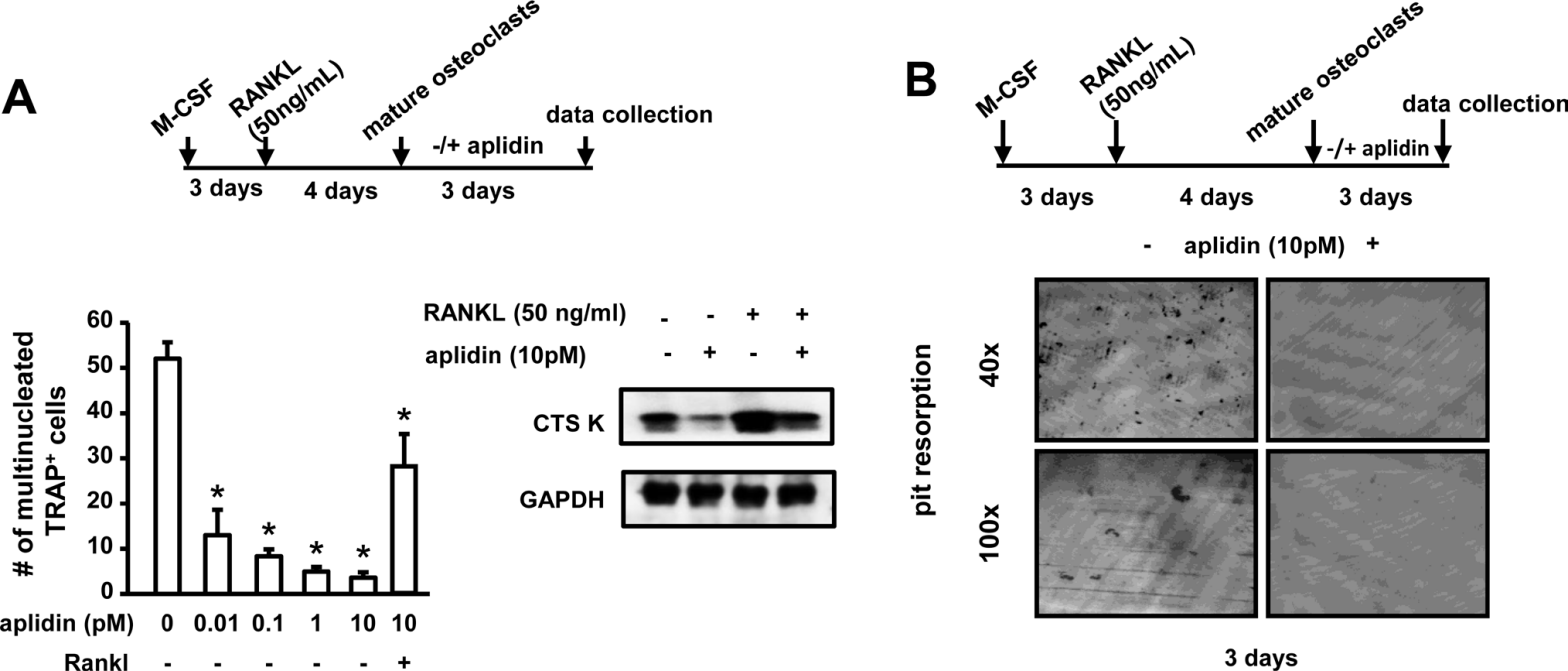
Aplidin decreases the number and activity of mature osteoclasts (**A**) Number of TRAP+ mature osteoclasts and protein expression of Cathepsin K after treatment with Aplidin for 3 days in the presence/absence of RANKL. (**B**) Resorption pits formed on bovine bone slices by mature osteoclasts treated with Aplidin for 3 days. Representative images out of 4 independent replicas are shown per each condition. Representative experiments out of two are shown (*n* = 4 per condition). Bars represent means ± SD. ^*^p < 0.05 Aplidin alone vs vehicle.

We then tested the effects of Aplidin on mature bone resorbing osteoclasts. Aplidin decreased the number of mature osteoclasts in a dose-dependent manner, and 10 pM Aplidin reduced the increase in Cathepsin K induced by RANKL in mature osteoclasts (Figure 5A). In addition, Aplidin, either alone and in combination with Dex and Btz, markedly decreased the number of resorption pits formed by mature osteoclasts plated on bovine bone slices. (Figure 5B and Supplementary Figure 1).

### Aplidin exhibits anti-myeloma and anti-resorptive activities in an *ex vivo* 3D bone organ model of multiple myeloma

**Figure 6:**
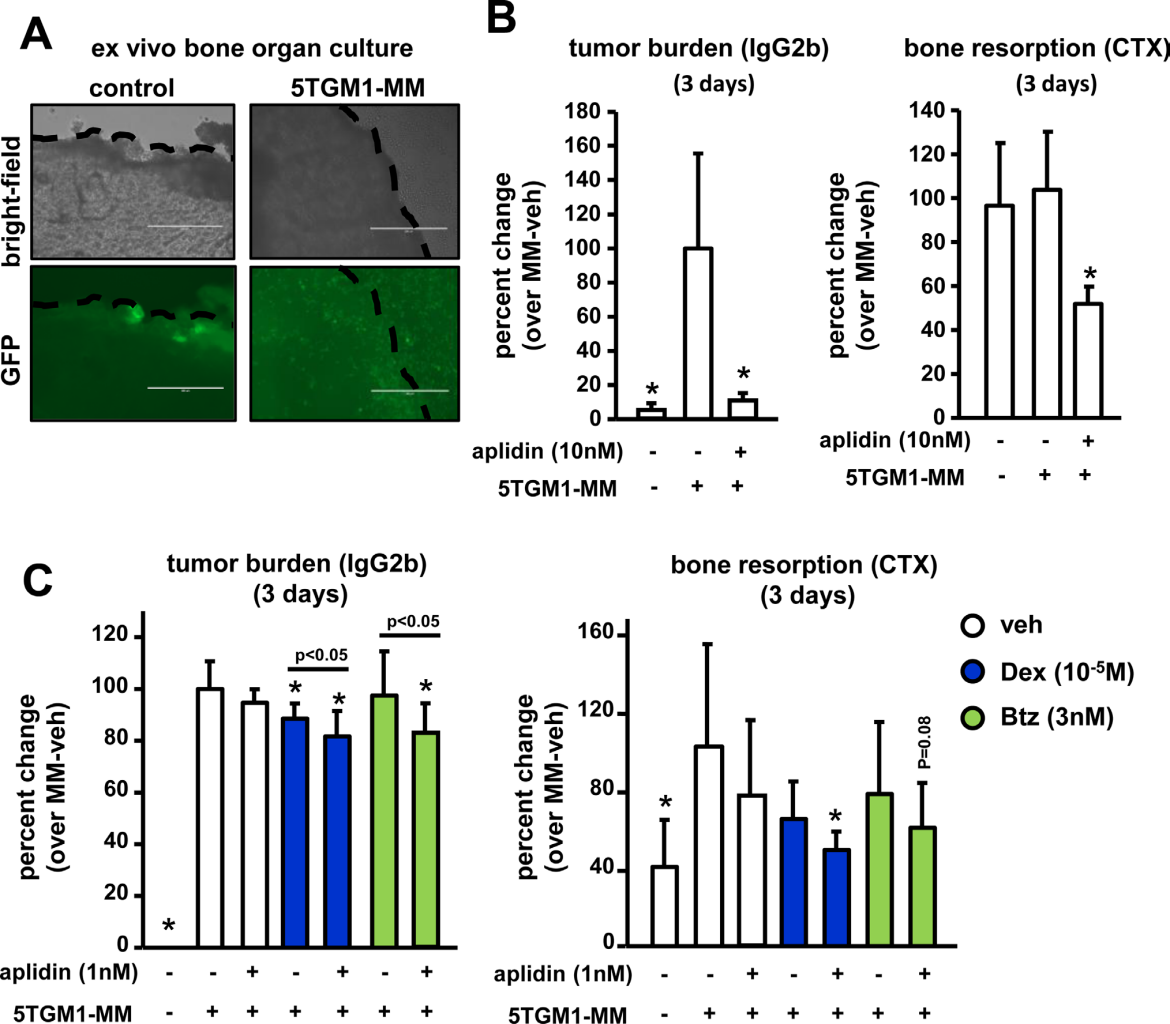
Aplidin decreases tumor growth and inhibits bone resorption in an *ex vivo* bone organ model of multiple myeloma The levels of the tumor growth marker IgG2b and the bone resorption marker CTX were evaluated in conditioned media collected from control bones and bones bearing 5TGM1 MM cells. (**A**) Bright field pictures show the piece of calvarial bone and GFP fluorescent images show 5TGM1-GFP MM cells within the bone. Bones bearing MM cells were cultured with/without Aplidin alone (**B**) or combined with Dex and Btz (**C**). IgG2b and CTX levels were determined after 3 days. Percent change was calculated over bones bearing MM cells and cultured under vehicle conditions. Combined results of two independent *ex vivo* studies are shown. 4-6 independent calvarial bones were used per condition/experiment. Bars represent means ± SD. ^*^p < 0.05 vs MM-veh; lines indicate *p* values < 0.05 comparing MM-Dex/Btz alone vs MM-Dex/Btz + Aplidin.

We next examined the effects of Aplidin on tumor growth and bone resorption using an *ex vivo* bone organ culture model of MM in which the three-dimensional organization and the cellular diversity of the MM/bone marrow niche are preserved, thus providing a physiologically relevant environment (Figure 6A) [13–16]. After 3 days of culture, conditioned media (CM) collected from calvarial bones bearing MM cells exhibited a 19-fold increase in levels of the tumor marker IgG2b compared to CM derived from control bones. Three days of treatment with 10 nM Aplidin reduced the levels of IgG2b by 60% in CM from bones bearing MM cells (Figure 6B). After 12 days of treatment, Aplidin completely blocked tumor growth, as IgG2b levels in CM from bones bearing MM cells treated with Aplidin were indistinguishable from those found in CM from control bones (Supplementary Figure 2A). In this particular experiment, we were not able to detect significant increases in the levels of the bone resorption marker collagen type 1 cross-linked C-telopeptide (CTX) in CM from bones bearing MM. However, treatment with 10 nM Aplidin for 3 days markedly decreased CTX to levels below those found in CM collected from control bones (Figure 6B). Further, CTX levels were undetectable in bones treated with 10nM Aplidin for 12 days (Supplementary Figure 2A). These findings are consistent with our *in vitro* studies showing decreased number of osteoclasts after Aplidin treatment, and support the notion that, in addition to its potent anti-MM properties, Aplidin also has the potential to block bone destruction by inhibiting bone resorption.

We next investigated in this model the effects of combinations of suboptimal doses of Aplidin, Dex, and Btz on tumor growth and bone resorption. Three days of treatment with 1 nM of Aplidin or Btz as single agents did not significantly decrease IgG2b levels in CM from bones bearing MM cells, whereas treatment with Dex alone modestly decreased MM growth. In contrast, combined administration of Aplidin and Dex or Aplidin and Btz significantly decreased tumor burden, demonstrating that Dex and Btz exhibit additive effects with Aplidin, enhancing its anti-MM properties (Figure 6C). After 12 days, 1nM Aplidin alone decreased tumor growth by 40%, and no additional significant decreases in MM cell burden occurred when combined with Dex or Btz (Supplementary Figure 2B).

CM from bones bearing MM and treated under vehicle conditions cells had increased CTX levels compared to control bones not bearing MM cells (Figure 6C). Treatment with Aplidin, Dex, or Btz alone did not alter the increased CTX levels in CM MM myeloma-bearing bones after 3 days. In contrast, treatment with combinations of Aplidin and Dex decreased CTX levels in CM from MM-bearing bones (Figure 6C). Co-administration of Aplidin and Btz also decreased CTX levels, however these results did not reach statistical significance (Figure 6C). After 12 days, Aplidin alone reduced CTX in bones bearing MM by 60%. Moreover, Aplidin in combination with Dex and Btz, completely prevented the increased bone resorption induced by MM cells, with CTX levels no different from those observed in CM from control bones (Supplementary Figure 2B).

## DISCUSSION

**Figure 7:**
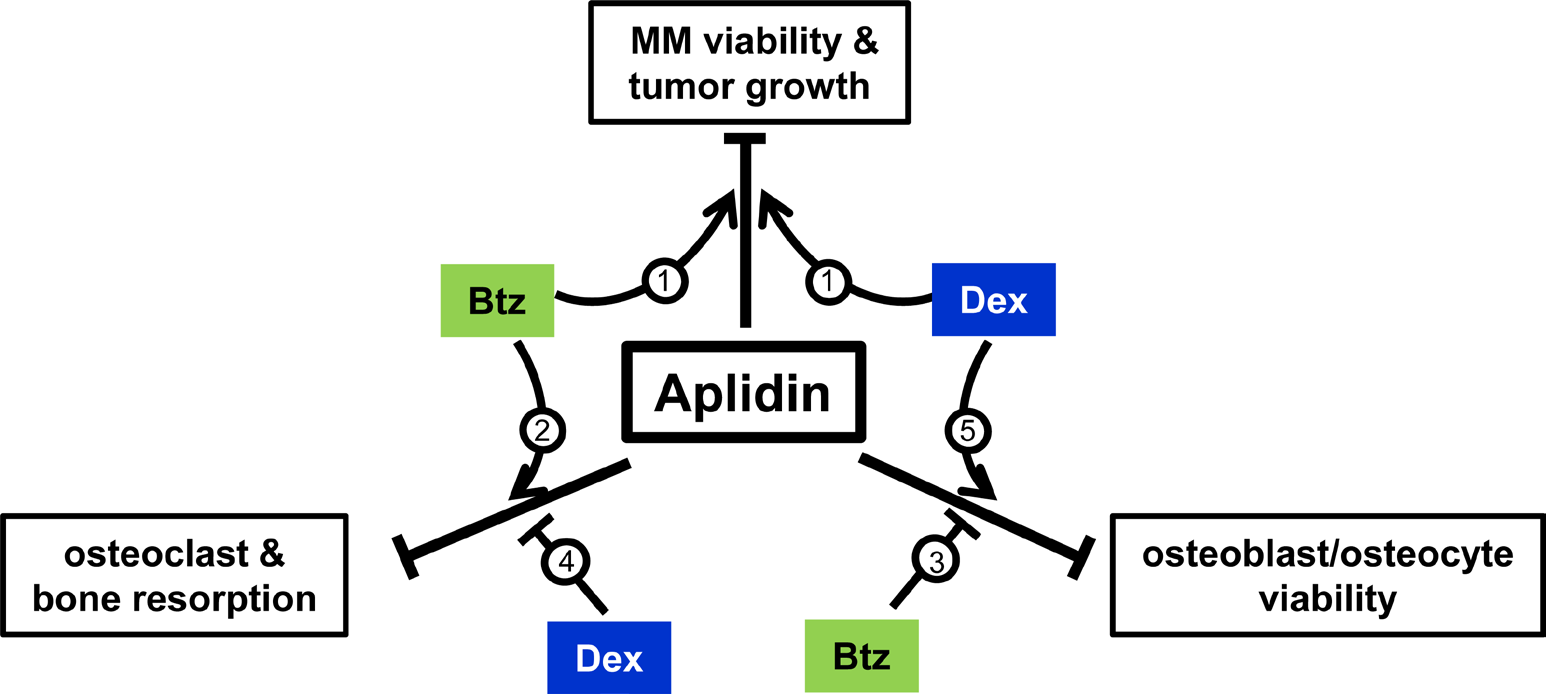
Working model Aplidin inhibits MM cell viability, decreases osteoblast/osteocyte viability, and inhibits osteoclast differentiation and function. Co-administration of Aplidin enhances the anti-MM activity of Btz and Dex (**1**). Further, when combined with Aplidin, Btz increases the anti-resorptive properties of Aplidin (**2**), and partially prevents the decreases in osteoblast/osteocyte viability and osteoblast function induced by Aplidin (**3**). In contrast, Dex increases bone resorption (**4**) and in combination with Aplidin further decreases osteoblasts/osteocyte viability (**5**). Thus, administration of Aplidin as a single agent has the potential to simultaneously inhibit tumor growth and prevent bone destruction by inhibiting bone resorption. Moreover, in combination with Btz, it could result in superior anti-MM and anti-resorptive activities than each agent alone, as well as ameliorate the deleterious effects of Aplidin on osteoblasts/osteocytes. Finally, although co-administration of Dex and Aplidin could also result in enhanced anti-MM effects, Dex may antagonize the anti-resorptive activity of Aplidin and further increase osteoblasts/osteocyte cell death.

Bone destruction is the hallmark of MM, with approximately 70% MM patients presenting with osteolytic lesions at the time of diagnosis. Despite the abundance of new drugs recently approved for the treatment of MM, the disease remains incurable and the skeletal consequences persist even after achievement of long-term tumor remission. Further, many anti-tumor treatments have adverse effects on bone (i.e. Dex, alkylators, long-term bisphosphonate administration), resulting in more rapid and severe bone destruction [5]. Thus, new more effective therapeutic approaches that improve anti-tumor responses and maintain bone health in patients with MM are needed. The results of this study demonstrate that Aplidin decreases MM growth and enhances the efficacy of other chemotherapy drugs (Btz and Dex) on MM cells (Figure 7). In addition, we found that Aplidin targets pre-osteoclasts and mature osteoclasts and potently inhibits their differentiation and bone resorptive activity. Further, Aplidin acts additively with Btz to inhibit MM cell-induced bone resorption in an *ex vivo* model of MM bone disease. The potent anti-resorptive activity of Aplidin found in our studies, together with its potent activity against MM cells, strongly suggest that treatment with Aplidin could simultaneously block MM growth and bone destruction in MM patients.

Our studies show for the first time that Aplidin exhibits a potent anti-resorptive activity through direct actions on osteoclast precursors and mature osteoclasts. These cells exhibited a very high sensitivity to Aplidin compared to MM cells and other bone cells (picomolar versus nanomolar doses). Aplidin decreased Cathepsin K protein expression in mature osteoclasts, and prevented osteoclast differentiation by blocking the induction of osteoclast differentiation markers (NFATc1, TRAP, MMP-9) and inhibiting the rapid ERK phosphorylation induced by RANKL signaling, key molecular events required for proper osteoclast formation and function [17]. This latter observation is in line with previous findings showing that Aplidin decreases ERK1/2 and ERK 5 phosphorylation in MM cells [9]. However, unlike in MM cells, Aplidin did not stimulate or altered RANKL-induced p38 phosphorylation, suggesting that Aplidin differentially regulates MAPK signaling pathways in non-tumor vs tumor cells.

High dose of glucocorticoids (GCs) is a frequent component of anti-MM therapy due to its ability to induce apoptosis of MM cells and capacity to enhance the efficacy of other chemotherapy drugs [18–20]. However, GCs cause rapid bone loss by increasing bone resorption, inducing osteoblast and osteocyte apoptosis, and reducing bone formation [21–23]. In contrast, Btz, also used as a frontline agent for the treatment of MM, can induce transient decreases in bone resorption and increase osteoblast number and bone formation markers [24]. Our *in vitro* results show that Aplidin counteracted the increase in osteoclasts differentiation induced by Dex, whereas in combination with Btz, it induced a more potent suppression of osteoclast differentiation and bone resorption than each agent alone. Together, these results show the potential of combining Aplidin with GCs or Btz for the treatment of MM, as these combination therapies could prevent tumor growth whilst reducing the bone loss induced by MM cells and mitigate the adverse effects of GCs on the skeleton.

Our proliferation and cell death studies in human and murine MM cells lines demonstrate that Aplidin exhibits anti-proliferative and pro-apoptotic activities in MM cells. In addition, combination of Aplidin with other anti-MM drugs induces better responses than each treatment alone, demonstrating that Aplidin enhances the anti-MM effects of Dex. This is consistent with previous findings showing that co-administration of Aplidin and Dex results in additive anti-MM effects [9], and with more recent results of phase III clinical trials demonstrating that patients with relapsed/refractory MM treated with combined combination of Aplidin and Dex had a significantly 35% lower risk of disease progression or death when compared to Dex alone (6). Further, Aplidin decreased the viability of JJN3 cells and enhanced their response to Btz. JJN3 cells have mutated p53, which can contribute to resistance to Btz and poor outcomes in MM patients [25]. Our findings are in line with a previous publication showing additive effects of Aplidin against MM cells when combined with Btz [9]. However, it remains unclear whether co-administration of Aplidin could overcome resistance to proteasome inhibitors and Dex. Together, these data suggest that Aplidin has a unique mechanism of action that does not interfere with pathways targeted by other anti-tumor drugs. In addition, our findings suggest that Aplidin could be effective against MM cells resistant to frequent components of anti-MM therapy and may help to overcome the drug resistance that patients develop over the course of treatment.

Treatment with Aplidin also induced cell death in osteocytes (via apoptosis), and osteoblasts, and enhanced the cell death induced by Dex in these cell types. Our results also suggest that co-administration of Aplidin and Dex leads to further decreases in tumor growth than each agent alone. However, this combination could further suppress osteoblast number in bones bearing MM. In contrast, we found that Btz alone did not affect osteoblast/osteocyte viability, but it partially prevented the increased osteocyte/osteoblast cell death induced by Aplidin. This is consistent with a previous report showing that Btz prevents osteocyte cell death induced by Dex and MM cells *in vitro*, and that patients treated with Btz exhibit higher numbers of viable osteocytes compared with those not treated with Btz [26]. Mechanistic studies revealed that these protective effects of Btz are due to inhibition of osteocyte apoptosis and autophagic cell death [26]. Similar to a previous report showing that Btz induced transient increases in osteoblast and circulating markers of bone formation [24], we found that Btz mitigated the reduction in osteoblast mineralization induced by Aplidin. Thus, combined administration of Aplidin and Btz could result in superior anti-MM activity, stronger suppression of bone resorption, and ameliorate the potential negative effects of Aplidin on bone formation. Future studies are warranted to examine the effects of Aplidin alone and in combination with current anti-MM drugs in *in vivo* models of MM-induced bone disease.

Our bone organ culture that reproduces the 3D-organization and the cellular diversity of the MM/bone marrow niche allowed us to examine the effect of Aplidin simultaneously on MM and bone cells, thereby providing a model closer to an *in vivo* scenario. Using this innovative approach, we demonstrated that Aplidin decreased MM cell growth and osteoclast-mediated bone resorption in bones bearing MM cells, and that suboptimal doses of Aplidin in combination with Dex and Btz have additive effects against MM cells and decrease resorption to a larger extent that each treatment alone. These *ex vivo* results demonstrate that Aplidin can inhibit bone resorption in the MM/bone marrow microenvironment and provide additional support for combining Aplidin with other anti-MM drugs for the treatment of MM. However, further studies are needed to evaluate if Aplidin also inhibits bone resorption and has combinatorial effects when co-administered with other anti-MM drugs in murine models of MM disease and MM patients.

In summary, our study increases the potential of Aplidin as a therapy for MM by demonstrating its effects on MM-induced bone disease. We show that Aplidin is a potent anti-resorptive agent that inhibits bone resorption by acting directly on osteoclast precursors and mature osteoclasts. Further, the anti-tumor and anti-resorptive properties of Aplidin are preserved and/or enhanced when combined with frequent components of anti-MM therapy. Together, these findings provide the framework for additional preclinical and clinical studies of this agent for the treatment of MM and its associated skeletal disease.

## MATERIALS AND METHODS

### Reagents

RPMI-1640, α-MEM, glutamine, penicillin, and streptomycin were purchased from Invitrogen Life Technologies (Grand Island, NY, USA). Aplidin was provided by PharmaMar S.A. (Madrid, Spain). Bortezomib (Btz) was purchased from Selleckchem.com (Houston, TX, USA), and Dexamethasone (Dex) from Sigma-Aldrich (St Louis, MO, USA). MTT from Invitrogen. Murine anti-p-ERK antibody (Cat. # 9101), anti-ERK antibody (Cat. #9102), anti-p-P38 antibody (Cat.# 9211) and anti-P38 antibody (Cat.#9212) and anti-GAPDH antibody (Cat.#2118) were purchased from Cell Signaling Technology (Danvers, MA, USA); β-ACTIN antibody (ab49900) from Abcam (Cambridge, MA, USA); anti-NFACT1 antibody (Cat.# sc-7294); anti-MMP9 (Cat.# sc-393859); anti-TRAP (Cat.# sc-28204); and anti-Cathepsin K antibody (Cat.# sc-6507) from Santa Cruz Biotechnology Inc. (Dallas, TX, USA); and anti-DC-STAMP antibody (Cat.# MABF39-1) from Millipore (Temecula, CA, USA). Human RANKL and M-CSF were purchased from R&D systems (Minneapolis, MN, USA); and bone slices for osteoclast resorption from Immunodiagnostic Systems Inc. (Gaithersburg, MD, USA).

### Cells and culture conditions

Human JJN3 and murine 5TGM1 MM cell lines, commonly used in our laboratory to model MM growth and bone disease in rodents [14, 30], were provided by N. Giuliani (University of Parma, Italy) and B. Oyajobi (University of Texas at San Antonio, TX, USA), respectively. Murine MLO-A5 osteocyte-like cells were provided by L. Bonewald (Indiana University, IN, USA) [27, 28]. OB-6 osteoblast-like cells were obtained from R. Jilka (University of Arkansas for Medical Sciences) [29]. MC3T3-subclone MC4 pre-osteoblastic cells were obtained from G.D. Roodman (Indiana University). 5TGM1 and JJN3 MM cells, MC4 pre-osteoblastic cells, OB-6 osteoblastic cells, and MLO-A5 osteocyte-like cells were cultured as previously published [13, 14, 30]. All cell lines were tested for morphology, gene expression profile, and tumorigenic capacity (MM cells) according to previous publications and proved to be mycoplasma free.

### Cell viability

5TGM1 and JJN3 MM cells (3 × 10^5^ cells/mL), and OB-6 osteoblastic cells and MLO-A5 osteocyte-like cells (1.5 × 10^4^ cell/cm^2^) were treated for 24 h and 48 hours with Aplidin (1–180 nM) in the presence or absence of Btz (3 nM) or Dex (10^−5^ M). Btz/Dex doses were selected based on previous studies showing that treatment with these concentrations decreases JJN3 and 5TGM1 cell viability by ~50% [30], thus allowing us the detection of combinatorial effects when co-administered with Aplidin. JJN3 MM cells and MLO-A5 osteocyte-like cells were pre-incubated with DEVD (100 nM) for 4 h and then treated with Aplidin 10nM for 48h. DEVD was refreshed every 24 h. DMSO and ethanol were used as controls for these experiments. After treatment, 5TGM1 and JJN3 MM cells, OB-6 osteoblastic cells, and MLO-A5 osteocyte-like cells were pelleted and resuspended in media. Cell death was quantified by Trypan blue uptake as described [14, 30], and cell viability was examined by MTT assays following protocols established by the manufacturer.

### Osteoblast differentiation

MC-4 and OB-6 osteoblastic cells (5000 cells/cm^2^) were cultured with osteogenic media (0.2 mM ascorbic acid, 10 mM β-glycerophosphate) as previously described [13, 14], and treated with Aplidin (1 and 10 nM), alone or in combination with Btz (3nM) every 3 days or once per week. Media were replaced every 2–3 days, and mineralization was determined by Alizarin Red staining and quantified as described before [13].

### CFU-GM colony formation

Murine non-adherent bone marrow cells (1 × 10^5^cells/culture) were cultured in α-MEM (Gibco, Grand Island, NY) containing 1.2% methylcellulose, 30% FBS, 1% deionized bovine serum albumin (BSA) (Sigma-Aldrich), and 100 ng/mL recombinant human GM-CSF (Immunex Corp., Seattle, WA, USA). The cells were plated in a volume of 1.0 mL in 35-mm culture dishes (Corning, New York, NY), as reported previously [31], and treated with or without Aplidin (5 pM and 50 pM). The number of colonies that formed were scored after 7 days of culture using an inverted microscope.

### Osteoclast formation from murine bone marrow

Bone marrow cells were flushed out from long bones of C57BL/6 mice and cultured in α-MEM containing 10% FBS overnight. Non-adherent cells were harvested and enriched for CD11b^+^ mononuclear cells as described previously [32]. To determine the effects of Aplidin on the growth of osteoclast precursors, CD11b^+^ cells were cultured in α-MEM containing 10% FBS plus 10 ng/mL of M-CSF for 3 days in the presence or absence of Aplidin (0.01–10 pM). To assess the effects of Aplidin on osteoclast differentiation, CD11b^+^ cells were cultured in α-MEM containing 10% FBS plus 10 ng/mL of M-CSF for 3 days and then treated with RANKL (50 ng/mL) for 4 days in the presence/absence of Aplidin (0.01–10pM), Btz (3 nM), and Dex (10^−7^ M). RANKL and other treatments were replenished every two days. Cells were stained for TRAP using a leukocyte acid phosphatase kit (Sigma-Aldrich), and TRAP-positive mononuclear cells and multinuclear cells (≥3 nuclei/cell) were scored microscopically after M-CSF for 3 days or after RANKL for 4 days respectively, as described previously [32].

### Isolation of mature osteoclasts from mouse bone marrow cultures and bone resorption assays

Bone marrow cells were flushed out from long bones of C57BL/6 mice and cultured in a 10-cm dish (2.5 × 10^7^ cells/dish) with M-CSF (10 ng/ml) for 3 days and RANKL (50 ng/ml) for 4 days as previously described [32]. Then, cells were incubated with trypsin EDTA (Corning) for 3 minutes to remove non-osteoclastic cells and enrich the concentration of mature osteoclasts in the cultures. Mature osteoclasts (1 × 10^4^/well in 96-well plates) were treated with or without Aplidin (0.1–10 pM) for 3 days in the presence/absence of RANKL (50 ng/ml). For bone resorption assays, 5 × 10^4^ mature osteoclasts were transferred to bovine slices and cultured with RANKL (50 ng/mL) and treated with Aplidin (0.1 and 10 pM) in the presence or absence of Btz (3 nM) and Dex (10^−7^ M) for 3 or 7 days. RANKL (50 ng/mL) was added every two days to the resorption assays.

### Western blot analysis

Protein lysates were prepared from osteoclast precursors undergoing differentiation and from mature osteoclasts as previously described [15]. Cell lysates (25 μg) were boiled in the presence of sodium dodecyl sulfate (SDS) sample buffer (0.5 M Tris–HCl, pH 6.8, 10% wt/vol SDS, 10% glycerol, 0.05% wt/vol bromophenol blue) for 5 minutes and subjected to electrophoresis on 4% to 20% SDS -PAGE (Bio-Rad Laboratories, Hercules, CA, USA). Proteins were transferred to nitrocellulose membranes using a semidry blotter (Bio-Rad) and incubated in blocking solution (5% nonfat dry milk in TBS containing 0.1% Tween-20) for 1 hour to reduce nonspecific binding. Membranes were washed extensively and immunoblots were performed using anti-p-ERK (1:1000), anti-ERK (1:1000), anti-p-P38 (1:1000), anti-P38 (1:1000), anti-NFACT1 (1:1000), anti-CATHEPSIN K (1:1000), anti-DC-STAMP (1:500), anti-GAPDH (1:2000), anti-MMP9 (1:1000), anti-TRAP (1:200), and anti-β-ACTIN (1:4000) antibodies followed by rabbit anti-goat or goat anti-mouse antibodies respectively, conjugated to horseradish peroxidase (1:2000) in 5% milk, Santa Cruz Biotechnology). Western blots were developed using an enhanced chemiluminescence detection assay following the manufacturer’s directions (Bio-Rad).

### *Ex vivo* bone organ cultures

Bone organ cultures were established as previously described [14]. Briefly, calvarial bones from 9–15 day-old mouse C57BL/6 pups were cut into 3.5 mm disks with a biopsy punch and placed into 24-well plates. 5 × 10^4^ 5TGM1 MM cells were added in 0.5 ml of RPMI medium supplemented with 10% fetal calf serum and antibiotics. Bones bearing MM cells were washed with PBS, transferred to a new plate, and treated with Aplidin (1 nM or 10 nM) in the presence or absence of Btz (3 nM) and Dex (10^−5^ M). Conditioned media was collected 3 and 12 days after treatments for assessment of tumor growth by IgG2b levels and bone resorption markers by C-terminal telopeptides of type I collagen (CTX). Half of the media was replaced every 3 days until day 7, when 100% of media was replaced with fresh culture media. Murine IgG2b concentrations in conditioned media were determined using commercially available enzyme-linked immunosorbent assays (ELISA) kits, according to manufacturer instructions (Bethyl Laboratories, Inc., Montgomery, TX, USA). CTX levels in conditioned media were quantified by ELISA (Immunodiagnostics Systems) as previously published [33].

### Statistics

Data were analyzed using SigmaPlot 12.0 (Systat Software, Inc., San Jose, CA, USA). All values are reported as means ± SD. Differences between treatment groups were evaluated using One-way ANOVA. *p* ≤ 0.05 was considered statistically significant.

### Study approval

Studies using animal samples (calvarial bones) were approved by the Indiana University School of Medicine’s IACUC.

## SUPPLEMENTARY MATERIALS FIGURES


