# Supplementary material for: Aplidin (plitidepsin) is a novel anti-myeloma agent with potent anti-resorptive activity mediated by direct effects on osteoclasts
Source: Oncotarget. 2019 Apr 12;10(28):2709–21. doi: 10.18632/oncotarget.26831 (PMC6505631; doi:10.18632/oncotarget.26831)
Supplement: Supplementary file 1 [file oncotarget-10-2709-s001.pdf]

Aplidin (plitidepsin) is a novel anti-myeloma agent with potent anti-resorptive activity mediated by direct effects on osteoclasts

SUPPLEMENTARY MATERIALS

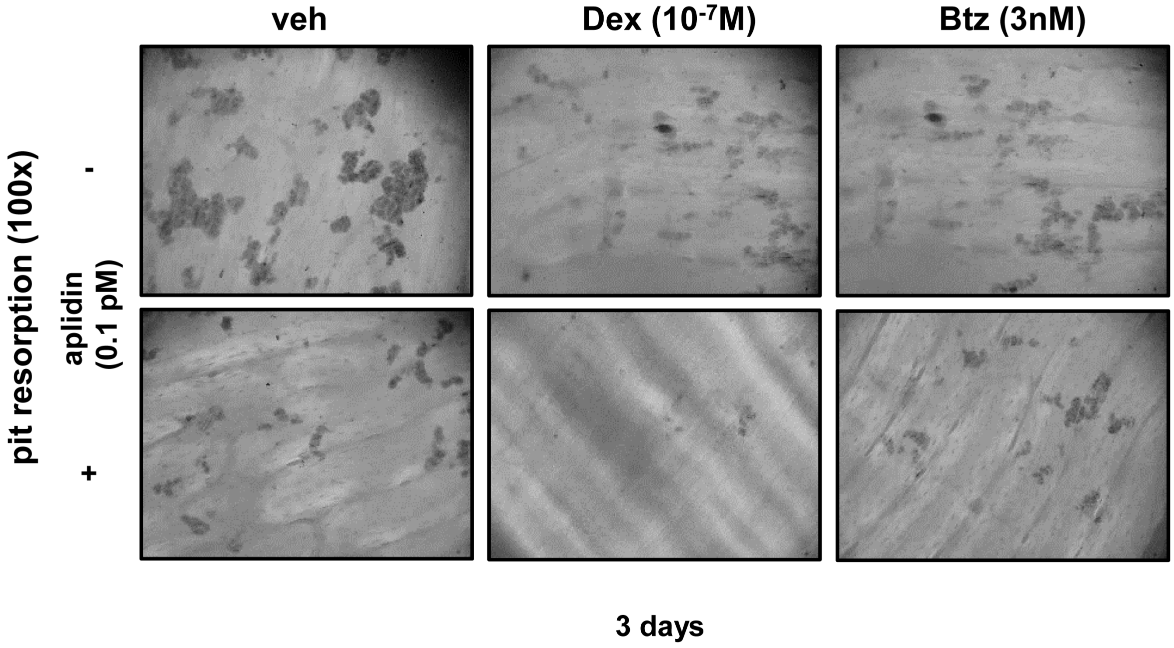

Supplementary Figure 1: Effects of Aplidin on osteoclastic bone resorption. Resorption pits formed on bovine bone slices by mature osteoclasts treated with Aplidin alone, or in combination with Dex or Btz for 3 days.

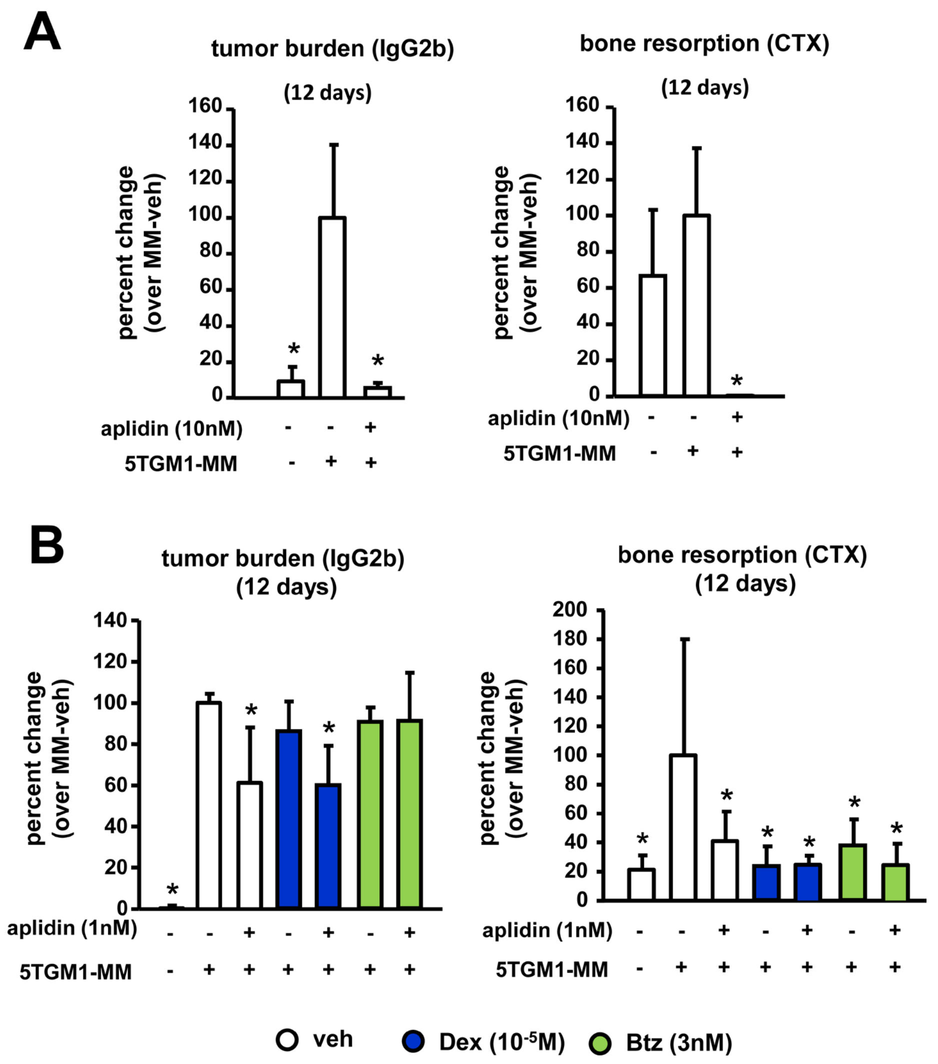

Supplementary Figure 2: Effects of long-term treatment with Aplidin on *ex vivo* MM-bone organ cultures. Bones bearing MM cells were cultured with/without Aplidin alone (**A**) or combined with Dex and Btz (**B**). IgG2b and CTX levels were determined after 12 days. 4–6 independent calvarial bones were used per condition. Percent change was calculated over bones bearing MM cells and cultured under vehicle conditions. Bars represent means ± SD. $^{*}p < 0.05$ vs MM-veh.
